# Effectiveness Analysis for Smart Construction Safety Technology (SCST) by Test Bed Operation on Small- and Medium-Sized Construction Sites

**DOI:** 10.3390/ijerph19095203

**Published:** 2022-04-25

**Authors:** Yong-Seon Kim, Jae Yun Lee, Young-Geun Yoon, Tae-Keun Oh

**Affiliations:** Department of Safety Engineering, Incheon National University, Incheon 22012, Korea; k94022@kosha.or.kr (Y.-S.K.); jaeyun.lee@daewooenc.com (J.Y.L.)

**Keywords:** construction, automation technology, smart construction safety technology (SCST), safety management, small- and medium-sized site

## Abstract

In the global construction industry, government policies have recently focused on smart construction technologies, such as those concerning the “smartization” of construction, improvements of productivity, and automation technologies. In addition, smart construction safety technologies (SCSTs) have been developed to ensure workers’ safety, under the initiative of the private sector. In regards to overseas occupational safety, wearable technologies have been developed for various types of industries, and the integrated platform developments needed to link them have become mainstream. In South Korea, individual companies are focusing on developing basic SCSTs and platforms for integrated control, aiming to prevent accidents in the construction field. The goal of this study was to identify the pros and cons of SCSTs through test bed operation and to derive improvement directions. Therefore, a test bed embedded with SCSTs was built and operated to provide effective safety management for small- and medium-sized sites exposed to fatal accidents. From analyzing the data from the test bed, it was found that it is difficult to change the tendencies of workers’ behaviors based solely on the introduction of SCSTs. This indicates that the effects of SCSTs are insignificant without the cooperation of workers. In addition, technical problems in field application were identified for each sensor and equipment, and the necessity, problems, and effectiveness of SCSTs were analyzed. As a result, both the installation and attachment types were found to be effective; however, workers avoided wearing certain attachment types. Based on the results derived through analysis of the pros and cons of SCSTs, the directions and guidelines were suggested for future use. This result can be used for future technology development directions, and policy establishment. Additionally, for the activation of SCSTs in the field, the cooperation of workers and the interest of managers remain essential factors, and improvements to the equipment are required.

## 1. Introduction

**Figure 1:**
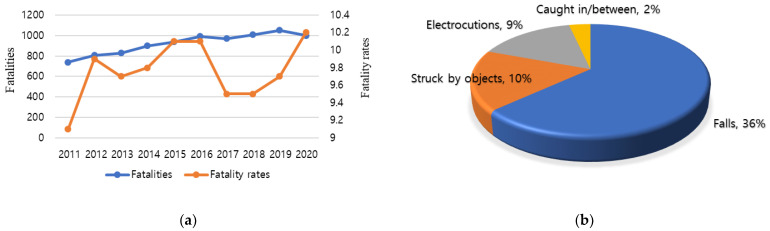
Construction accident trends reported by the Occupational Safety and Health Administration (OSHA) in 2021. (**a**) Construction fatalities and fatality rates *; * Fatality rates: Fatalities per 100,000 Full-Time-Equivalent works for the private-sector construction industry. (**b**) Four main causes of workplace deaths.

Globally, changes have recently been demanded from the construction industry owing to social trends, such as aging workers, a lack of skilled manpower, and a reduction in working hours. The death toll from occupational accidents in construction is more than twice that from other industries; correspondingly, there is a need for the construction industry to solve its chronic safety problems [1]. According to the US Occupational Safety and Health Administration (OSHA), there were 1008 fatalities in the construction industry in 2020 (Figure 1a), accounting for approximately 20% of the total industry. Of these, 351 people died from fall accidents. OSHA also ranks the “Fatal Four” hazards accounting for the majority of injuries and deaths based on data from the U.S. Bureau of Labor Statistics, i.e., those accounting for over 60% of all construction worker deaths. These four major risks are falls, being struck by objects, electrocution, and being caught in/between objects (Figure 1b) [2].

According to the 2016 European Small Business Annual Report and 2015 US Small and Medium Business Administration statistics, the employment ratios of small- and medium-sized enterprises (SMEs) in the construction industry are 87.6% and 82.7%, respectively [3,4]. As SMEs represent over 80% of construction workers, improving safety management at construction sites implies that SMEs must play important roles in protecting their workers. Factors affecting safety performance in construction SMEs include the number of workers, limited availability of skilled manpower, lack of experience and training, resource availability, unsafe attitudes, business size, construction cost, and time [5]. At smaller sites, the safety management system for construction workers may not be implemented properly, as these are relatively free from government regulations and duties; therefore, safety management compliance depends on the internal atmosphere of site safety managers and workers [6]. Moreover, as there are many safety rules, it is difficult to identify the appropriate safety instructions and communicate them to the right people and/or the right workplace.

Considering the numerous safety guidelines for construction sites, even workers or inexperienced managers may not be aware of (or may ignore) certain safety requirements during construction work [7]. In general, the safety awareness of SMEs is insufficient. As a result, problems such as neglecting to comply with safety rules or misunderstandings of guidelines frequently occur, leading to accidents. Moreover, although the participation of safety experts is required to design a safety plan, SMEs claim that they do not have the necessary funds to hire such professional personnel. To overcome this, SMEs need new safety policies and improved smart construction safety technology (SCST). It is necessary to introduce new strategies, such as those based on SCSTs.

### 1.1. Literature Review

Smart construction technology (SCT), also known as intelligent construction technology, denotes a fully networked system of sensors, displays, and computing elements with embedded intelligence and advanced digital applications [8]. SCT has received increasing attention and application in the construction industry worldwide. To take advantage of SCT for construction engineering and management (CEM), various studies on the development of numerous applications for SCT have been conducted and published in the last 10 years [9]. Some researchers have focused on the economic benefits of SCT [2,3], whereas others have focused on technological developments [9]. Regarding the strengths of SCT for CEM, it has recently become apparent that CEM should include construction-related tasks, processes, issues, and human collaboration elements related to SCT [10]; nevertheless, the application of SCT in CEM has inherent complexity. Therefore, various aspects of SCTs are being considered in this context, such as the cost and time savings related to SCTs, improvements in the productivity and safety of construction projects, promotion of location detection for construction elements [8], risk mitigation and digitalization, and computer vision approaches [11].

In the past decade, SCT technologies have been introduced to the construction industry [12]. For example, Jiang et al. [13] proposed a cyber-physical-system-based on-site safety management system for monitoring human and mechanical positioning, environmental conditions, and other risks in the field. By defining the concept of digital skin, Edirisinghe [8] emphasized using intelligence and (correspondingly) building future smart construction sites by integrating hardware components, communication technologies and software, and middleware/applications. Pan and Zhang [12] published a review of artificial intelligence (AI) implementations, benefits, and research for existing CEM approaches, and suggested essential directions for future research to facilitate the use of AI in CEM. Štefanihc and Stankovski [14] identified and summarized emerging smart construction applications, including those concerning construction site management, construction monitoring, early warning systems, resource, and asset management, and work safety.

Many scholars have discussed the need for digitalization in the architecture, engineering, and construction industries in terms of Industry 4.0 and/or Construction 4.0 [15,16]. Industry 4.0 and/or Construction 4.0 represents a much broader field than SCT, and includes topics such as construction automation, robotics, and modularization. Štefanihc and Stankovski [14] briefly described emerging smart construction applications in areas such as construction monitoring, work safety, and early disaster warnings. Most existing studies have reviewed the possibilities from a relatively low/narrow perspective, rather than considering a comprehensive view of the technology. Vovchuk et al. [17] suggested that sound waves can be used effectively to extinguish fires in petroleum and petroleum products and can be used in future development of robots for fire suppression at construction sites. Pięta et al. [18] used a smart Internet of Things (IoT)-based multi-domain model to simulate tourist behavior and congestion avoidance in a theme park. Belka et al. [19] presented a concept of a visitor support system for a distributed entertainment park based on IoT and big data analytics, which is flexible and scalable based on the implementation and integration of modern IT networks, tracking, and monitoring technologies, decision support systems, and big data concepts. Płaza and Pawlik [20] provided a mechanism for multi-criteria content classification and behavioral profiling approaches, considering service level, cost, customer satisfaction, average processing time, abandonment rate, average latency, and occupancy as key performance indicators. Although there are technological developments in various industries using IoT, it is in the early stages in construction sites.

### 1.2. Application Limit to Small- and Medium-Sized Construction Sites

In most countries, SCSTs are primarily applied to large construction companies and public institutions. However, there is an urgent need to establish SCSTs for small- and medium-sized construction sites. In another way, it is necessary to develop a “smart construction safety system” model suitable for the size of each respective construction site. Currently, in the “4th Industrial Era” at various construction sites, SCTs such as Building Information Management (BIM), drones, Information and Communications Technology (ICT)-based technologies, and SCSTs have been introduced and used (or demonstrated) for the safety management of workers. Although it remains difficult to fully utilize smart technologies, academia, clients, and construction companies have researched and developed on-site application methods and field applications.

In South Korea, large construction companies and public institutions are beginning to introduce technologies such as those based on the IoT, sensors, and wearables (e.g., in 2020), but in small- and medium-sized construction sites, the application of SCSTs remains limited, owing to costs and a lack of information. Thus, there is a growing gap. Currently, building an SCST at a construction site requires professional measuring instruments and expensive sensors. Accordingly, the initial costs for introducing smart safety management systems at small- and medium-sized construction sites are high. In addition, as the versatility of the equipment in the field is weak, it can be inefficient to apply it to various sites; therefore, an easy and favorable smart safety management system is needed. Accordingly, it is necessary to apply SCSTs to construction sites from the perspectives of design, construction, and safety management, and to verify their effectiveness. Since SCSTs are in their infancy, there is little data on the concept, effectiveness, and problems of applied technologies. In this regard, this study built and operated a test bed with representative smart safety equipment and sensors and then analyzed the necessity of SCSTs, convenience, and effectiveness from the field perspective. In addition, the activation plans and development direction were presented at the current technology level.

## 2. Smart Construction Technology (SCT)

### 2.1. Concept & Development of SCT and Smart Construction Safety Technology (SCST)

SCTs can be defined as all types of technologies for innovating and improving productivity based on providing information sharing and connectivity for all participants in all stages of construction work, such as in planning, design, construction, maintenance, and demolition, e.g., by utilizing “4th Industrial Revolution” element technologies [21]. The concept of a SCT was created to innovate construction productivity and enhance safety, and it is defined as a technology that combines traditional construction technology with 4th Industrial Revolution technologies such as BIM, drones, robots, the IoT, big data, and AI. SCTs innovatively changed the typical paradigm of the construction industry by applying 4th Industrial Revolution technologies to various stages of the industry, such as design, construction, and maintenance [16].

In the design stage, the optimal design is performed in a 3D-based virtual space, and simultaneously, integrated planning and management activities can be conducted while considering the construction and operations from the design stage [22]. During the construction stage, parts are manufactured and constructed in a factory without being affected by weather or civil complaints, and even unskilled personnel can cooperate with various sensors and devices to enable high-level work, leading to equipment automation and increased intelligence. In the maintenance stage, facility information is collected in real time, and various smart technologies are used to create an environment for conducting scientific and objective analyses of the conditions and performance of facilities.

**Figure 2:**
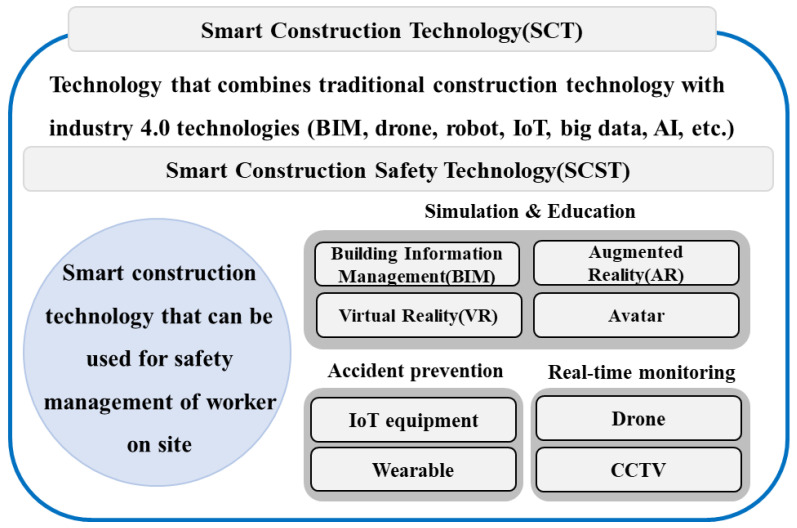
Definitions of SCT and SCSTs (including closed-circuit television (CCTV), building information management (BIM), augmented reality (AR), virtual reality (VR), artificial intelligence (AI), and Internet of Things (IoT)).

Among SCTs, the definitions or concepts of SCSTs are very diverse, and consensus has not yet been established [9,23,24]. Figure 2 illustrates the basic concept of SCST by comparing SCTs and SCSTs, to which 4th Industrial Revolution technologies have been applied. In general, SCST remains a transitional technology until the completion of SCT, and includes technologies for education, accident prevention, and real-time monitoring, aiming to ensure the safety of on-site workers during construction.

Globally, research in the field of technologies for recognizing risk factors for workers at construction sites is increasing; in this context, wearable smart device technology supports construction site safety inspections. In general, technologies for recognizing risk factors provide, e.g., 3D content for support, information using augmented reality (AR), and an IoT platform for connecting inspectors and control systems [8]. Smart safety technology was initially applied mainly to gas and oil plants but is gradually being expanded to other industries.

**Figure 3:**
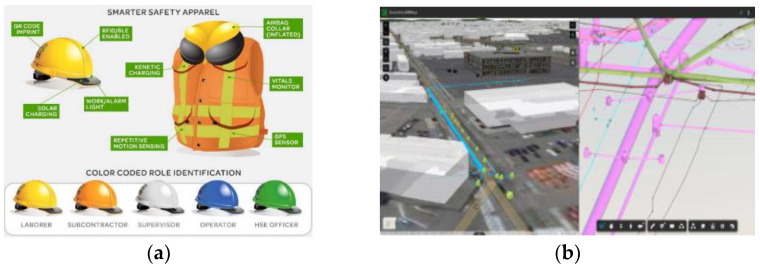
Major development directions of SCST. (**a**) Wearable safety technology for human condition safety. (**b**) Safety check using AR/VR technology of AUTODESK.

Countries such as the United States, Australia, and Japan have developed big data technologies, IoT technologies, and wearable equipment to improve safety at construction sites. For example, in 2016, American International Group (AIG) strategically invested in a human condition safety startup company that used IoT technology to build a safe environment for workers, and provided on-site environmental information analysis (Figure 3a). Synaptor in Australia has developed a big data system for preventing safety accidents at construction sites, analyzing data produced at construction sites in real time, and warning workers.

As shown in Figure 3b, AUTODESK released the AUTODESK FORGE AR/Virtual Reality (VR) Toolkit, which visualizes 3D BIM, including design drawings and detailed information on the web, and can be observed with AR/VR equipment. After matching a 3D BIM model stored in the cloud and at the inspection site by a special target with AR/VR equipment, it visualizes information on risk factors during a safety inspection, records additional discovered risk factors, and suggests functions that can be stored. Epson released “Moverio smart glasses” with Wi-Fi and a Bluetooth communication module that could be connected to the manager or control program. The system comprised a GPS sensor and inertial measurement unit sensor for location recognition and behavior detection for workers, and safety glasses for providing 3D content and AR functions for inspection targets. RealWear developed hmt-1, a tablet PC that could be installed in a hard hat. Notably, htm-1 was connected to a control system. Accordingly, the necessary information could be visualized through the smart glasses when observing the main inspection location, and video and audio were shared by experienced technicians (“mentors”) by utilizing the control system and/or a video call function.

With regard to smart fabrics consisting of wearables and bio-monitoring clothing for understanding the physical and mental states of a person, the US and Japan have provided original technological capabilities centered on major companies, such as DuPont, Toray, Teijin, and Toyobo. Europe is leading the world market for high-tech technologies, such as smart textiles and smart clothing, and has focused on securing source technologies, such as advanced electronic textiles and medical textiles, for ICT and convergence. In Europe, the European Union has jointly invested in creating a smart fabric interactive textile cluster, and has developed smart textile technologies by focusing on unit projects such as myHeart, Biotex, Proetex, Stella, Ofseth, Context, Mermoth, and Systex.

### 2.2. Review of SCST Cases

**Figure 4:**
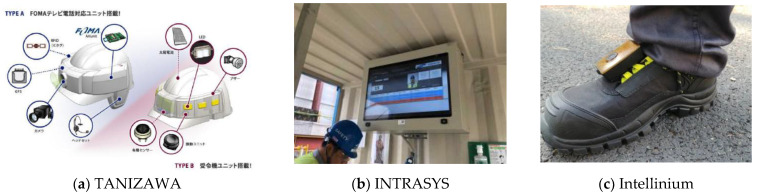
Smart construction safety equipment in major countries.

The application cases for SCSTs, mainly in foreign countries, were investigated and are summarized in Table 1; the main equipment is shown in Figure 4. SCSTs have been found to be similar to each other worldwide, and it is generally understood that there are many companies converging the various element technologies. However, overseas, there are differences in performing various functions simultaneously, such as loading a large number of element technologies into one safety device or commercializing them for product development and convenience in work. Nevertheless, technologies have been developed to support the recognition of the different risk factors for various sites for the safety of construction site workers, provide a smart eye for managers, recognize harmful environments and biometric information of workers, and provide active personal protective equipment. Moreover, technologies have been developed for minimizing unsafe behaviors from recognized risk factors, in addition to risk dynamic risk assessment programs, worker decision-making analysis and optimization programs, and on-site customized training content.

### 2.3. Major Smart Safety Sensors & Equipment in South Korea

Although governments such as the USA, the UK, and Korea have implemented quality and safety management systems (such as regularly supervising sites with high accident rates and giving disadvantages when evaluating public construction projects according to the results), in general, the smaller the site, the more difficult regulation is in reality. In addition, many accidents occur owing to a lack of implementation of the appropriate system, and/or the low safety awareness of workers. One effective safety management method for overcoming the difficult conditions in small- and medium-sized sites is to introduce innovative solutions, such as those based on SCSTs. As there is a high vulnerability to accidents at these sites, owing to the absence of a professional safety manager, it is important to provide methodologies and supporting tools. From this perspective, smart safety equipment has been developed, and can mainly be divided into worker-attached and on-site installation types. The operation principles and main functions are summarized in Table 2 and Table 3.

Currently, such equipment has reached a level usable for the safety management of construction sites based on advanced technologies, such as cyber physical systems, artificial intelligence-based deep learning, and IoT, which are the fundamental technologies of the 4th Industrial Revolution. Although interest in the 4th Industrial Revolution and the willingness to utilize its corresponding technologies are high, the implementation strategies for specific technology application plans and effects analysis remain insufficient. The reasons for the lack of technological innovation include insufficient private technology development efforts, experts, government policy direction, and related systems. For the construction industry to respond to the 4th Industrial Revolution, governments must actively create an environment to encourage the application of advanced technologies in the construction industry. Based on this data, construction systems with deep learning and risk-level predictions should be built and ensured to support systematic and logical analyses and response solutions in the construction safety field, including accident prediction models and/or integrated construction safety control systems for site safety.

## 3. Test Bed Selection and Operation

### 3.1. Test Bed Selection

As mentioned, accidents at construction sites have not decreased, but rather have stagnated, and accidents such as falls and being struck by objects continue to occur. In particular, greater numbers of accidents have occurred at small- and medium-sized sites because the construction costs and time are limited, and managers and safety management personnel may be absent. In this context, the application of SCSTs can alleviate the problems associated with small- and medium-sized sites. However, SCSTs have generally not been distributed to small- and medium-sized sites owing to cost problems, and the effectiveness of such SCSTs has not been verified.

Therefore, in this study, three locations were chosen for representative types of structures among construction sites. A test bed was operated, its effectiveness was confirmed by the confirmation and analysis of the results, and problems and improvement plans were derived according to the current technology levels.

**Figure 5:**
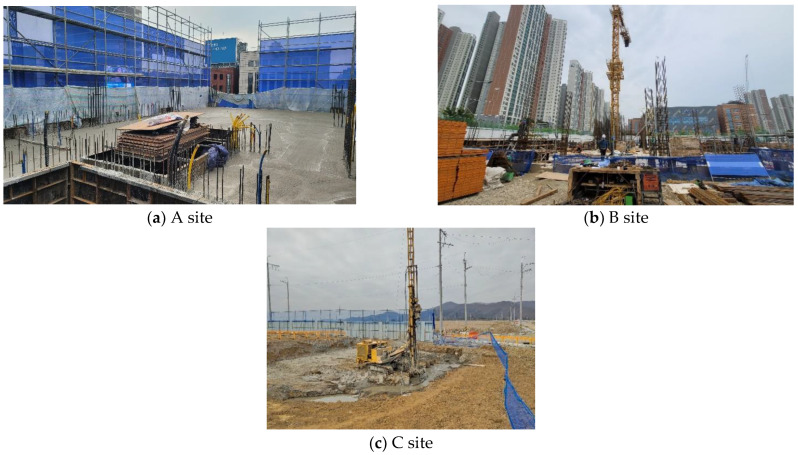
Site images of the selected test beds.

The main site of this project was set as a target for small- and medium-sized sites with less than $12 million. Considering the accident type, (1) a site with a small site area and high floor height (A site) and (2) sites with a large area and low floor height (B and C sites) were selected. Front views of the three test beds are shown in Figure 5.

### 3.2. Test Bed Operating Equipment Configuration

**Figure 6:**
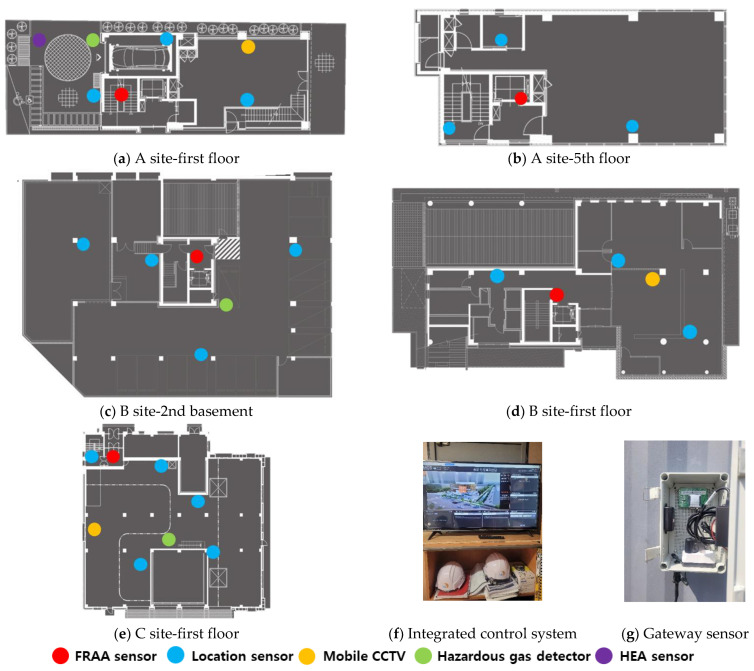
Site-specific sensor installation location and basic installation equipment.

The test bed was operated from July to October 2021. The equipment and associated systems, including site visit and installation plans, were built in June 2021. The number of sensors and equipment were selected based on location identification, aiming to minimize blind spots by selecting proper equipment/sensors for SCSTs. The study also aimed to prevent and manage major accidents, such as falls, being struck by objects, and being caught in/between objects potentially occurring at each site based on site visits and analyses of drawings. To ensure the consistency of data collection, equipment installation was conducted simultaneously at all three sites, and the initial settings were established through installation and testing. After the initial setting of the equipment and systems, data were collected remotely from July to October 2021 (4 months) using an integrated control system, and maintenance was performed once a month. The site information, installation equipment, and quantities for the three sites selected as test beds are listed in Table 4, and the installation locations are shown in Figure 6.

### 3.3. Data Type Derived by SCST Equipment and System

**Figure 7:**
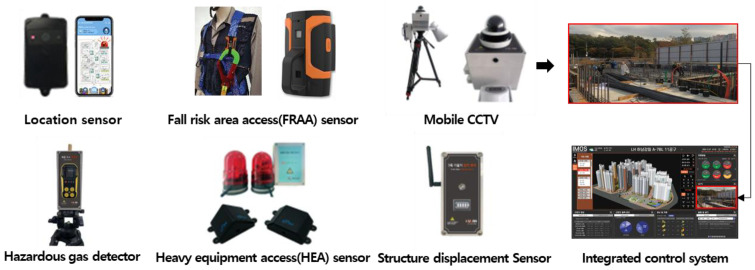
Types of SCSTs used in the test beds.

The SCSTs applied to the test bed are shown in Figure 7. There were differences in the log data recorded by the equipment, and a log data format for the system (i.e., for the introduced equipment) was examined to explore the various analysis possibilities using the collected log data.

**Figure 8:**
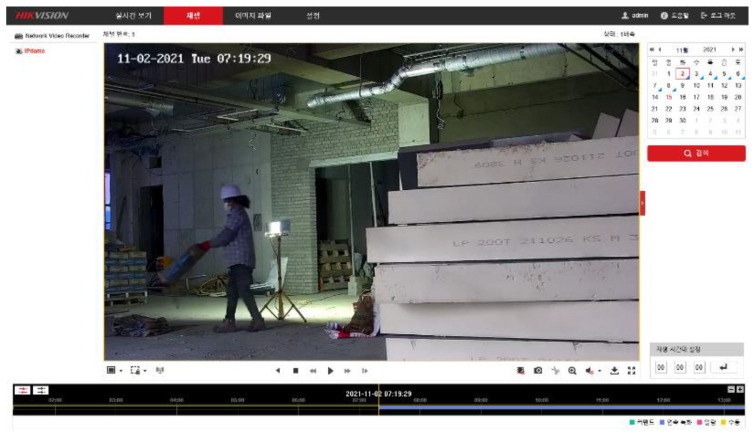
CCTV raw data.

Location sensors were used to confirm workers’ locations, and data were recorded for entry and exit times, construction companies, names, types (entry, general, risk areas), areas (floor), and locations. The Fall Risk Area Access (FRAA), Heavy Equipment Access (HEA), and structure displacement sensors had the formats. The data concerned the time, location, equipment name, anti-work type (detailed location), detailed work type (fastening, approach, and range), and output format fused thereto. The access sensors for confirming an approach to a fall risk area or heavy equipment when in operation for accidents, such as falls or being struck by heavy equipment, had a format in which the transceiver transmitted data at set times. For example, when a worker approached, the data was transmitted and received, and a recognition alarm or the like was transmitted according to the type of data. When the structure displacement sensor data exceeded a set threshold, an event was generated and stored. A hazardous gas detector can measure and record the oxygen, temperature, humidity, hydrogen sulfide, carbon monoxide, and methane every minute, making it possible to check these in the installed areas and locations. In this study, however, data were not recorded for hazardous gases during the test bed operating period; therefore, they were excluded from this part of the analysis. Mobile closed-circuit television (CCTV) was used to check real-time video recorded with date and time information, as shown in Figure 8.

## 4. SCST Performance Analysis

### 4.1. Sensor Data Analysis

Table 5 shows the location sensor data collected during the working hours of a worker at site A among the test bed sites for one day. From analyzing the data from the location sensor, general content, such as the commuting time of the worker, work area by time, working route, and lunch time, can be observed. Moreover, in terms of safety management, the workers’ working hours can correspondingly be adjusted. In addition, if the data are associated with a hazardous gas detector, it is possible to check whether the proper rest time is observed according to the temperature and humidity. In addition, it can check access to areas where hazardous gases (hydrogen sulfide, carbon monoxide, etc.) are generated during cold-weather concrete curing.

However, because the location sensor records thousands of data points per worker per day, it is considered that providing real-time confirmation in connection with other equipment is more appropriate than a post-data analysis of the number of output personnel.

**Figure 9:**
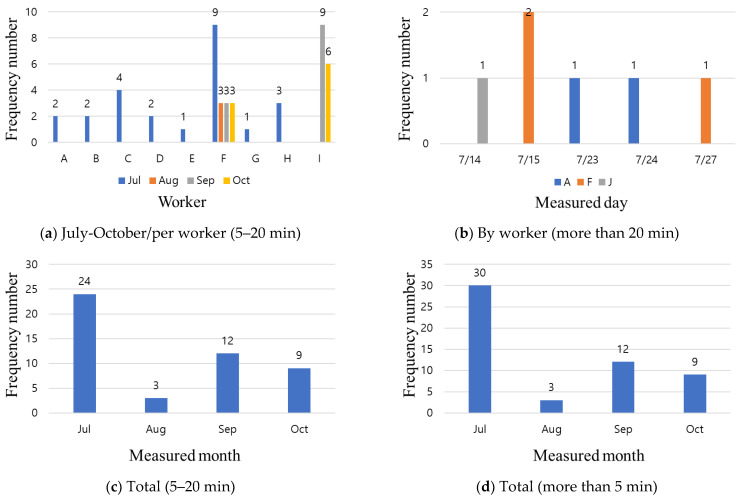
Analysis of fall risk area access (FRAA) sensor data for A site.

**Figure 10:**
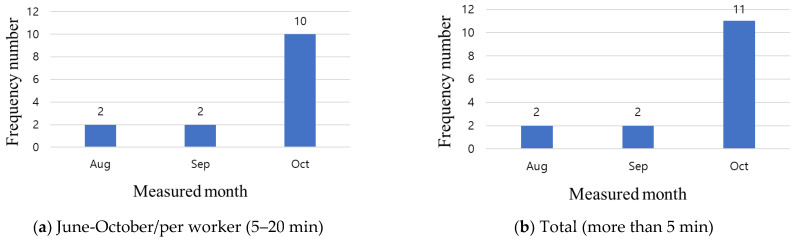
Analysis of FRAA sensor data for B site.

In this study, the fall risk area (including other risk areas) was set up according to the test bed site-specific process, and workers working at the location wore a smart safety belt (FRAA sensor) to prevent fall hazards; four months of data were collected and analyzed. Site A recorded access to the fall risk area for 1612 cases over those four months. Notably, these records do not mean that a dangerous situation occurred 1612 times, as they included cases recorded while moving to a different floor or other area; therefore, a separate standard needed to be prepared. Therefore, as shown in Figure 9, from analyzing the data of the workers staying in the fall risk area for more than 5 min, there are 54 cases, and among them, six cases represent workers staying for more than 20 min. In the case of Site B, no records are measured at the beginning of the process, and some data are measured as the structure floor increased, i.e., as the process progressed. A total of 64 pieces of data are measured, and as shown in Figure 10, four cases of staying for more than 5 min and one case of working for more than 20 min can be confirmed. The case of site C is similar to that of site B, but 196 approaches are detected in the four months as the floor height increases without basement construction. However, none of these jobs last longer than 5 min. In general, the problem with detecting the approach to the fall risk area is that when workers’ movements are within the transmission/reception range of the FRAA sensor, alarm, and log data are generated. As a result, for the three sites, if workers stay in the hazardous area for more than 5 min, they are considered to be working, but there is no decreasing trend. In general, it is possible to check for dangerous work by installing multiple mobile CCTVs, but considering the budgets of small- and medium-sized sites, this approach is somewhat limited.

**Figure 11:**
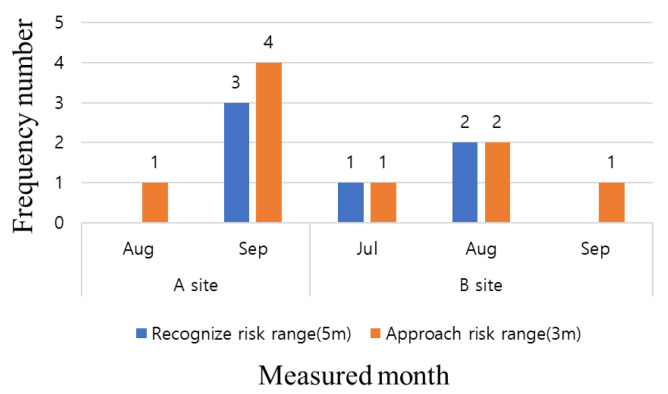
Analysis of heavy equipment access (HEA) sensor data for test beds.

The HEA sensor used in this study transmitted a recognition-approach-danger alert according to distances of 5 m, 3 m, and 1 m between the worker and heavy equipment, and when such an event occurred, a log was recorded in the control system. To confirm the effectiveness of the HEA sensor, it was installed in heavy equipment, such as forklifts and dump trucks, and the log records of the number of approaches were analyzed, as shown in Figure 11. As shown in Figure 11, some logs of heavy equipment access are recorded at Sites A and B, but no records are observed at Site C. At Sites A and B, where the logs are recorded, workers approach the heavy equipment at up to 3 m, and do not approach the hazard warning stage (1 m). In the case of HEA sensors, there are not many events; therefore, it is difficult to understand the decreasing trend in the data. Heavy equipment is used intermittently, i.e., only when there is a job, and the number of uses is low. From another perspective, if a worker approaches heavy equipment and an alarm is generated, workers who are slightly farther away from the site may recognize the warning, i.e., that heavy equipment is operating in the vicinity.

**Figure 12:**
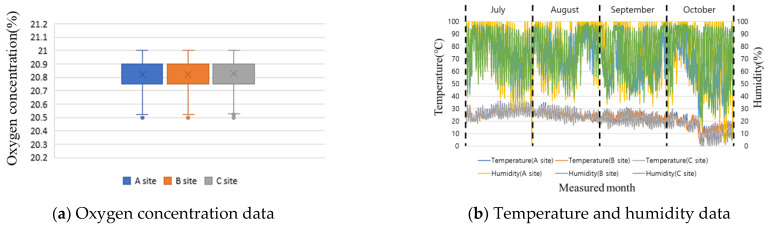
Analysis of hazardous gas detector data for test beds.

Figure 12a shows the results of measuring the oxygen concentration for the four months at the three sites using a hazardous gas detector. From a functional perspective, the minimum standard is generally measured once per minute; however, in this study, in consideration of the amount of data to be stored, measurements were conducted once every 5 min. Working at an oxygen concentration of 18% or less can lead to death owing to asphyxiation, depending on the lack of oxygen. However, as can be seen from Figure 12a, there is little variation in the oxygen concentrations measured at the three sites, and the values range from 20.5% to 21%. Sites A and C have a structure without a basement, and in the case of site B, although there is a basement, there is no welding work in the closed space during the test bed period. Oxygen concentration measurements can be used by placing the detector in the relevant space when working indoors in the basement. In the case of humidity, there are no restrictions, such as stopping work depending on the degree; in contrast, when the temperature is over 35 °C, it is stipulated to stop outdoor work. The temperature and humidity values measured at the three sites for four months are shown in Figure 12b. The temperature is above 35 °C at site A for 10 days in July and 1 day in August. In the case of site B, a temperature of 35 °C or higher is measured for two days, and for site C, it is measured for 10 days.

An alarm was generated according to the threshold value of the hazardous gas detector, and it was confirmed that the outdoor work was stopped according to the aforementioned regulations, followed by being changed mainly to indoor work. In small- and medium-sized sites, workers may die of heatstroke owing to managers’ lack of attention to temperature. In addition, a lack of rest owing to high temperatures can lead to deterioration of health conditions, such as anemia and dehydration. As such, hazardous gas detector data collection and dissemination should be used.

**Figure 13:**
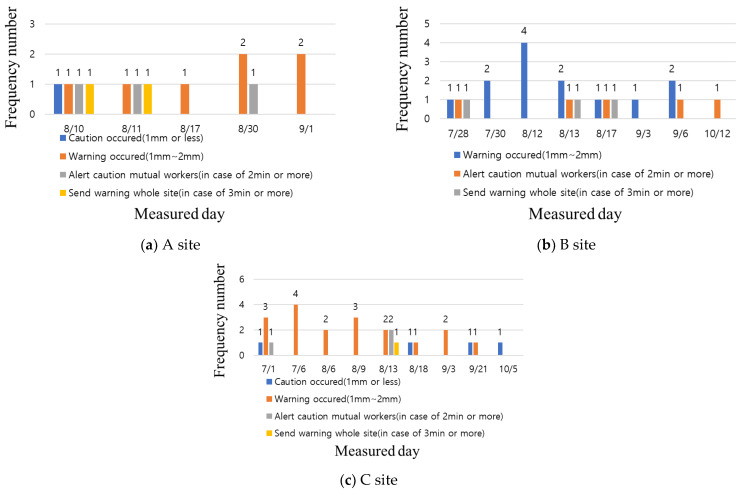
Analysis of structure displacement sensor data for test beds.

The structure displacement sensor was installed and operated on an external scaffold and formwork. The alarm criteria according to the displacement were as follows: caution—1 mm or less (one-off), warning—1 mm–2 mm (one-off), warning to workers—1 mm–2 mm (more than 2 min), and total field alarm—1 mm–2 mm (more than 3 min). Thus, the alarm consisted of four stages. From Figure 13, it can be confirmed that a Stage 4 alarm occurs at sites A and C. From checking with the mobile CCTV and on-site manager, it was confirmed that no accidents such as collapses of the formwork or scaffolding occurred. Thus, it is considered that this displacement is due to the loads of workers and materials applying pressure or loads on scaffolding owing to concrete pouring. Although the frequency of structural collapse is low, it leads to a major accident when it occurs; therefore, it is possible to inform workers of the situation and instruct evacuation in real time through automatic monitoring of the structure displacement sensor. This is expected to be useful in accident prevention.

**Figure 14:**
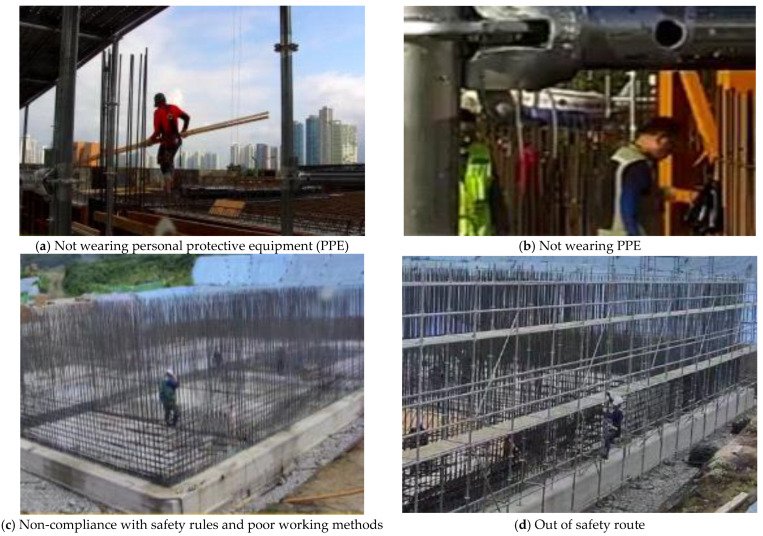
Problems of small- and medium-sized sites identified through CCTV.

The problems identified at the small- and medium-sized sites include non-compliance with safety rules, such as not wearing personal protective equipment (PPE), such as hard hats and safety belts and failing to fasten safety rings when working at heights. In addition, there is unrelated worker access to fall hazard areas and equipment, departure from movement routes, and smoking. Figure 14 shows an image of the test bed site, as confirmed through the mobile CCTV during the field operation. Most workers generally observe the above rules, but as some workers continue to not follow the safety rules, the need for guidance is highlighted.

**Figure 15:**
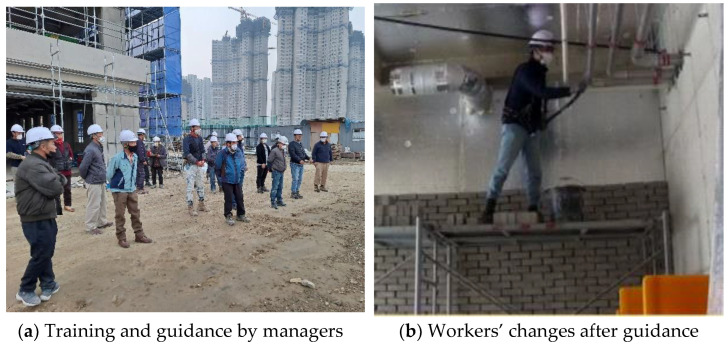
Changes in worker behavior through education and instruction.

For workers not wearing PPE with consistency and not following safety rules, we visited the site periodically to educate them regarding SCSTs, as shown in Figure 15a. The SCST education was conducted periodically before the start of work in September. Workers were educated that wearing PPE and compliance with safety rules could be checked in real time through mobile CCTVs at the site. In addition, efforts were made to improve the safety atmosphere at the site by educating managers to place CCTVs in hazardous areas on the same day, and to broadcast frequently using the mobile CCTVs during work hours. As a result of the periodic guidance, workers recognized that the manager was checking real-time with the mobile CCTV and improved their usage of PPE, as shown in Figure 15b, except for lunch and break times. In addition, problems such as poor working methods and deviations from the movement path were considerably reduced. Thus, if workers are made aware of errors by guidance and real-time confirmation, they can solve the chronic and typical problems at small- and medium-sized sites, e.g., not wearing PPE, poor working methods, and compliance with the movement routes, thereby preventing main accidents such as falls and being struck by objects. As a result, to maximize the performance of mobile CCTVs, training, and real-time guidance for workers by managers is essential.

In this study, seven different types of SCSTs were well utilized in the field (location sensor, FRAA sensor, HEA sensor, hazardous gas detector, structure displacement sensor, mobile CCTV, and integrated control system). Nevertheless, several prerequisites were derived, as follows. Most importantly, workers should reduce their reluctance to SCSTs and actively wear smart equipment, such as safety belts, on their own. In addition, it is necessary to eliminate blind spots by deploying a large number of location sensors, or by expanding the transmission/reception range when on-site situations are not available. In addition, it is necessary to install one or more CCTVs within the available budget, and to properly operate SCSTs through the placement of managers in charge of real-time guidance.

In addition, a deep learning analysis was conducted using video data from the mobile CCTVs; this has high potential for future development and is expected to be useful for small- and medium-sized sites. Image analysis methods can be divided into image classification, object detection, and image segmentation, and deep learning research on images has been actively conducted since the introduction of convolutional neural networks.

**Figure 16:**
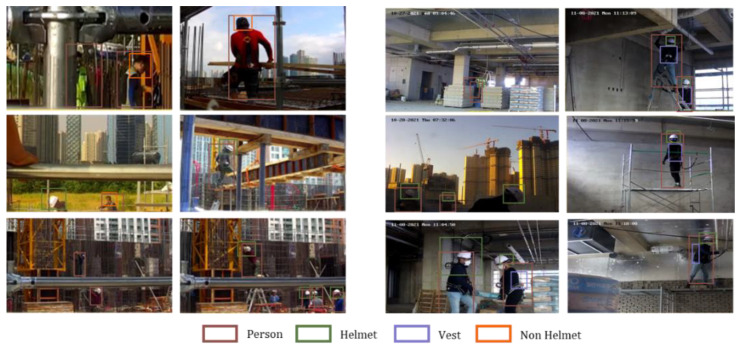
Analysis result of image sample using YOLO v2.

In this study, sample images collected during the test bed operation were used. Using the YOLO v2 model, a commercial deep learning algorithm supported by MATLAB, transfer learning was used to provide information on workers, safety helmets, and safety belts. The model was trained, and object recognition and tracking were performed using the developed model, as shown in Figure 16. As a result, the workers and safety helmets have object recognition rates of 70% to 80%, and tracking is generally good; however, in the case of safety belts, the rate is 50% to 60%, indicating that the recognition rate falls when using back and side views. At the current technology level, object detection performance for safety belts is not ready for the field application, but it can be overcome through the development of deep learning. Deep learning algorithms for video analysis are continuously developing, and SCST providers and image analysis companies are collaborating to develop intelligent CCTVs. Moreover, research on detecting unsafe behaviors of workers (rather than simply recognizing and tracking objects) is actively being conducted, and as the level of technology development increases, the utilization of mobile CCTVs is expected to increase further.

However, as the costs for supplying CCTVs with video deep learning are much higher than those for basic mobile CCTVs, it is also necessary to develop a deep learning model that focuses on specific accidents and lowers the unit price for introduction to small- and medium-sized sites.

### 4.2. Limitations and Improvement Plan of SCST after Test Bed Operation

As SCSTs are only being applied at some sites in large enterprises as pioneer programs, relatively few small- and medium-sized sites have introduced them, so there is a lack of analysis results. In this context, it is necessary to clearly understand current technology levels and field problems. Thus, after the test bed operations, the problems from the perspective of use in the field were analyzed based on the current technical level of SCSTs, and an improvement plan was derived to increase usability.

It was found that attachable-type SCSTs have common problems in terms of portability, convenience, and weight; moreover, there are often battery problems corresponding to the type of installation. In addition, the active participation of workers and guidance and/or supervision are prerequisites for these technologies to be activated in the field. Accordingly, in addition to analyzing common issues, technical, and field problems were analyzed for each piece of equipment, and improvement plans were derived for SCST activation. The results are as follows.

From the technical viewpoint of the location sensor, the problem is that the current level of technology is not high; therefore, log data are generally omitted during data collection, the battery rapidly loses power during operation, and additional heat is generated. When using a hazardous-access sensor by adjusting the approach distance, only the presence of the approach can be identified; there is no standard for whether it is actually a hazardous situation. In addition, when a dangerous area is set on a moving route, a log is continuously recorded and an alarm is generated, but the occurrence of the alarm is not necessarily linked to the dangerous situation. From a field perspective, one problem is that if many location sensors are installed, the cost increases, so small- and medium-sized sites with small budgets may have to leave blind spots for location identification. Therefore, it is difficult to detect when a worker moves to a space where a location sensor is not installed. Regarding other problems, in the case of smartphone-based sensors, the power is often turned off, and in the case of attachment-type sensors, it may cause inconvenience during work.

To solve these problems and increase usability, we propose the following. First, to improve the reliability of data collection (the most basic tasks), high interest from managers and the active participation of workers are required. As it is difficult to expect workers’ voluntary participation owing to the nature of small- and medium-sized sites, it is necessary to encourage worker participation through active management by managers.

Because worker participation is generally low for attachable products, it is necessary to introduce a smartphone-based location sensor to solve work obstructions along with accompanying technology developments. It also seems appropriate to reduce costs by eliminating blind spots and reducing the number of sensors while determining the locations of workers by increasing the sensing distance. When a location sensor is used as a hazardous-access sensor, the expected danger zone, according to the process table, can be set in advance, and the sensor can be appropriately placed. When an alarm occurs while a worker is approaching, it should be necessary for the worker to cancel the alarm after confirming with the manager. In addition, when a worker stays in a risk zone for more than 5 min, it should be classified as a dangerous operation, and improvements such as linking with the CCTV or checking directly with the manager should be necessary.

One problem in the operation of smart safety belts when checking for FRAAs is that workers must turn on the equipment when starting work, and it is necessary to install a separate beacon for transmission and reception at the construction site to check the results in the control system. At small- and medium-sized sites, workers change locations frequently, and the information entered in the system must be updated each time. In addition, because the smart safety belt is an attachment-type sensor, the participation of workers is required. If the smart safety belt is distributed to all workers, the budget increases excessively, so it is necessary to identify workers who work at a height in advance, and then intensively manage them by providing equipment. In addition, smart seatbelts can be checked in the control system by recording logs regarding recognition, caution, alarms, and confirmations of fastening, but it is believed that more efficient management will be possible if they are linked with mobile CCTVs. Currently, such systems work only when the equipment is turned on by the worker, but in the future, technological improvements are needed to automatically turn on the equipment when approaching a fall risk area. Thus, even if there is no worker participation, utilization is expected to be high.

The CCTVs to which IoT technology is applied are mainly of the movable installation type, and the problems identified after operation are as follows. A current CCTV operates individually without being linked to location and FRAA sensors, and there is a problem in that it is necessary to visually check the occurrence of an event from time to time during monitoring. In addition, the power sources for currently used mobile CCTVs can be divided into wired types and external batteries. In the case of the wired type, power must be connected over a long distance, which may interfere with workers’ movement. In the case of an external battery, there is a problem in that there is a need for a person in charge of charging and/or replacing. In addition, it may not be installed at the location of hazardous work on the same day (there is no person in charge), and in the case of small- and medium-sized sites, it is difficult to allocate a dedicated person in charge of such management and monitoring. Therefore, these systems are currently used mainly for work process checks, and not for safety management purposes. Mobile CCTVs allow for real-time confirmation of hazardous areas and work; therefore, it is easy to manage safety, such as by providing guidance.

From a technical perspective, the battery type is recommended because wired cables interfere with workflows. In the field, and under the active activities of the person in charge, it is necessary to install CCTVs at the locations of hazardous work (work at height, welding, etc.), monitor them using an integrated control system, and charge or replace the batteries after the work is finished. The effectiveness of mobile CCTV is expected to increase when used in conjunction with location and FRAA sensors. Currently, AI monitoring using deep learning is being actively developed; therefore, it is expected to be used more effectively for small- and medium-sized construction sites without managers in the future.

The hazardous gas detector has the same problems as the battery, as it has the same installation type as the mobile CCTV. As for the other problems, no hazardous gases are commonly generated except in special work, so it can generally only be used for measuring oxygen, temperature, and humidity. Currently, hazardous gas detectors are divided into two types: those attached to a structure, and those using a support. If there is a specific task (retaining concrete in winter and working in a confined space), they tend to only be used temporarily; therefore, it is appropriate to use the mobile type. Therefore, depending on the type of hazardous-gas detection sensor, it is expected that it can be used to classify, e.g., oxygen levels for sealing work, temperature, and humidity for hot and cold weather work, and hazardous gases for concrete curing.

An HEA sensor generates an alarm according to the initially set distance through transmission and reception between a sensor installed in the heavy equipment and equipment (attachable equipment or smartphone) carried by the worker. There is a corresponding technical problem in that it is difficult to distinguish between related and unrelated workers. The problem from the field side is that in the case of small- and medium-sized sites, the use of heavy equipment is generally concentrated in a specific period, so the utilization is low, and the manager must frequently install and dismantle the sensors owing to the frequently changed heavy equipment, making management difficult. As an improvement measure, the battery type can be improved so that it can be used with vehicle power, and equipment that operates based on a smartphone is recommended so that it can be operated even without an attached accessory. In addition, technological improvements are required to enable the same alarm when approaching from the rear side as well as when approaching from the side. In addition, the manager must prevent accidents in advance via linking with a mobile CCTV, e.g., by providing immediate guidance in a dangerous situation.

When installing a structure displacement sensor on-site, events may not occur because the allowable range is set low to prevent work interruptions owing to frequent alarms. Generally, the collapse of formwork or scaffolding occurs suddenly when the threshold is exceeded, so it may be difficult to detect only by measuring minute displacements. To solve this problem, the risk should be identified before collapse by preparing an actual risk threshold standard for the displacement slope that changes from the level at which one threshold is currently set. In addition, it is necessary to ensure the consistency of displacement measurements through periodic calibration.

An integrated control system has different functions depending on the company that develops and distributes the smart construction safety equipment, and thus may have little compatibility with other products. As the SCST market grows, it is necessary to construct common guidelines. Therefore, it is necessary to support the development of integrated modules so that SCSTs can be mutually compatible under the leadership of government-affiliated agencies in charge of safety.

**Figure 17:**
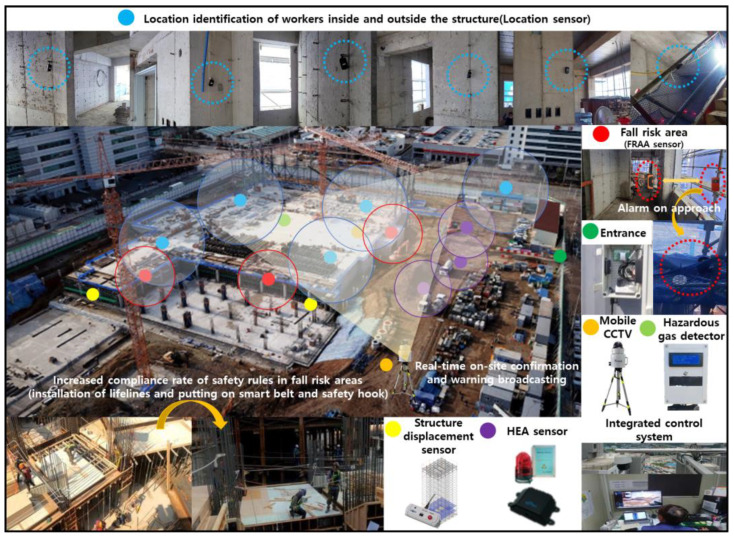
SCST installation and operation plan in small- and medium-sized sites.

In this study, SCSTs were introduced at small- and medium-sized sites, and problems and improvement measures were presented. If SCSTs are properly improved, as shown in Figure 17, the locations of workers, reductions in access to risk areas, and compliance with safety rules can be well established.

### 4.3. Recommendations for SCST from Test Bed Operation

The effectiveness of SCSTs for safety management was analyzed based on the strengths and weaknesses of each equipment and sensor, field application limits, and possible potential according to the test bed operation. As described above, there was a problem in deriving reasonable results based on the data because it was difficult to obtain meaningful data owing to the lack of active participation of workers in the test bed at small- and medium-sized sites. Therefore, a value analysis was conducted, focusing on the possibility of solving the problems and developing potential for the introduction of SCSTs. However, in the case of introducing all SCSTs, a large budget is required, even for small- and medium-sized sites. Therefore, the equipment expected to be effective in safety management through introduction to small- and medium-sized construction sites was classified into high, medium, and low categories according to necessity, cost, usability, convenience, and scalability. If necessary, the data were derived through interviews with field workers.

The combination of recommended technologies may vary depending on the site conditions, and it is necessary to consider the approximate budget for the introduction of such technologies. Based on the “high” and “medium” categories in Table 6, a recommended combination can be derived based on disaster type and construction cost, and the approximate budget can be analyzed. Most of the installation types are classified as “high,” and the attachment types are classified as “medium” or “low,” owing to the negative perceptions of workers.

## 5. Conclusions

Overall, small- and medium-sized sites have higher mortality rates, so efforts to reduce these rates are required. Moreover, there is a tendency for workers at such sites to have more accidents owing to poor site conditions, as well as undesirable construction costs, periods of work, and absences of safety managers. To minimize this, it is necessary to introduce SCSTs. From this perspective, this study built and operated a test bed at three sites, and conducted on-site verification of the smart safety integrated control system to be directly applied to the safety management of construction sites. The conclusions of this study can be summarized as follows.

1. Introducing SCSTs associated with incentives will likely lead to corresponding achievements, such as increases in workers’ compliance with safety rules and the creation of a safe culture.
2. A location sensor can be used for real-time location checks and commuting time checks, but it is considered difficult to use for safety management at the current technology level. Even if log data is analyzed, hundreds of data are recorded per worker per day, and location data omissions occur due to technology limitations and lack of workers’ participation; therefore, there is a limit to checking and managing the data after the record. Therefore, it can be effective to identify workers’ locations and manage associated with a mobile CCTV in real time at the current level.
3. An FRAA sensor generates an alarm and saves data if the movement of workers is within the transmission/reception distance of a risk zone approach sensor, similar to the functionality of the location sensor. However, there is a lack of quantitative criteria for determining whether workers are working in a risk area or simply moving. If the FRAA sensor and mobile CCTV are connected, it is easier to identify actual dangerous work, but because it is difficult to deploy many mobile CCTVs with high unit prices, it is possible to effectively manage workers with a stay period of 5 min or more according to standards.
4. In the case of a heavy equipment approach, an entire site alarm did not occur from the test bedsite and only the alarm according to the approach and recognition occurred. Owing to the characteristics of small- and medium-sized sites, heavy equipment is often detected in a single shot. Accordingly, installation and dismantling of HEA sensors may occur frequently; therefore, management by the site manager is required.
5. The hazardous gas detector was analyzed only for oxygen, temperature, and humidity information based on the absence of harmful gases owing to the construction characteristics and seasonal requirements. In August, the temperature was recorded as over 35 °C several times, but it was necessary to link with the mobile CCTV to determine whether a break time was actually given.
6. Structure displacement sensors for scaffolding and formwork may not experience any events during the test bed operation period. In particular, the displacement value is often set to be low; thus, events occur more rarely. However, when an event occurs, an alarm is sent according to the standard; thus, workers working in the vicinity can recognize it, and the effectiveness is judged to be high.
7. Even if a mobile CCTV was installed, there were many cases of workers not wearing protective gear and/or non-compliance with safety rules before training and guidance. In this context, a mobile CCTV is judged to be highly useful for safety management because it can be installed in an appropriate location, and the worker can be viewed and guided in real time if the manager is interested. In addition, if the recognition accuracy of safety belts can increase through the development of deep neural networks, mobile CCTV will also be more effective.
8. The SCST market is now in its infancy, and it is necessary to identify problems by applying SCSTs to various sites and to improve the technology, e.g., by reflecting the opinions of the site. In the case of attachable equipment, improvement is required so that it does not interfere with workers’ work, e.g., through lighter weights. In addition, institutional improvements and publicity should accompany the activation of SCSTs at the government and industrial complex levels. Insofar as the proposed recommendations, they have been analyzed based on the current technology levels. Moreover, the need for the introduction of SCSTs has been highlighted. It is highly likely that technology improvements from suppliers will proceed quickly, so there is a need to improve such technology levels through continuous research.
9. The pros and cons and combinations for each SCST are expected to contribute to setting the development direction of SCSTs and selecting SCSTs in small- and medium-sized sites.

## Figures and Tables

**Table 1:** Smart construction safety technology (SCST) companies and equipment in various countries.

Company Name	SCST
Wearable Technologies Limited (UK)	- Local hazard warning, vehicle proximity warning - Noise measurement, gas alarm - Vehicle proximity warning
TANIZAWA (Japan)	- Video shooting, real-time screen sharing - Location identification, voice communication, brain wave detection
INTRASYS (Singapore)	- Confirmation of completion of iris recognition and safety training
RealWear (USA)	- Video shooting, real-time screen sharing, voice communication
EXCELLENT WEBWORLD (USA)	- Location identification, collision prevention by around view monitor - Temperature, heart rate, harmful gas measurement, crisis situation notification
Intellinium (France)	- Crisis alert, Morse code transmission

**Table 2:** Major smart safety equipment with attachment type.

Attachment Equipment		Fundament and Method of Operation	Main Function
Location sensor	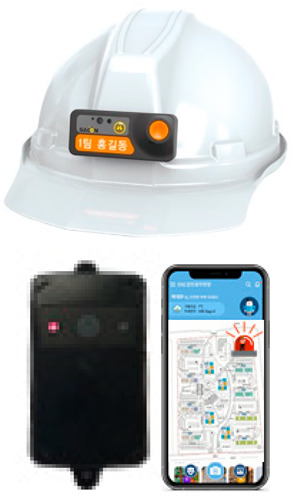	- Bluetooth Low Energy (BLE) or Bluetooth type - In conjunction with the sensor placed in the hazardous area, precautionary measures for approaching workers in the hazardous area	- Identifying the status of workers by location - Receiving information in hazardous areas, confined work, and heavy equipment work - In the event of an accident, information propagation to surrounding areas and managers - Identification of the status of workers by linking the control system - Check chin strap removal
Fall risk area access sensor (smart belt, safety hook)	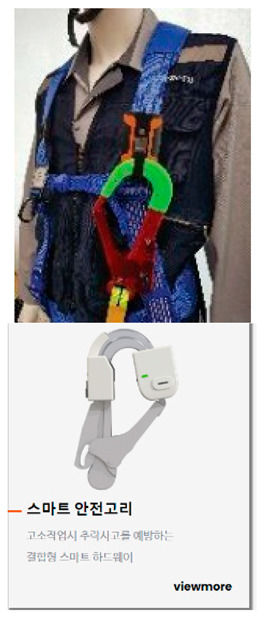	- BLE or Bluetooth type in building, Long-Term Evolution (LTE) outside - Interlocking wearable equipment and management app - In conjunction with the speaker installed in the fall risk area, a warning is generated when it is not fastened in the fall risk area. - Judging the presence of the worker at height engaged with the safety ring, location, & altitude.	- Checking the approach, the high-altitude work section, and whether the safety hook is fastened - Identifying the access time to hazardous are, number of times. - Detection of worker position and altitude
Heavy equipment access sensor	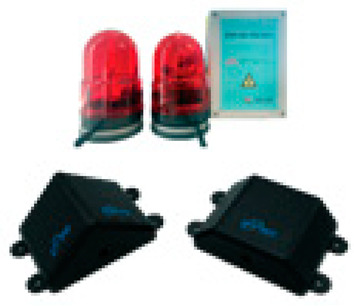	- LTE CAT M.1 type - Alarm when approaching workers by communication between ultrasonic sensor and worker’s mobile (Distance 1–5 m) - Radar utilization, peripheral approach detection, operator collision prevention. - Step-by-step warning by distance by detecting obstacles	- Measurement of time and frequency of heavy machinery access - Alarm broadcasting of equipment/worker access

**Table 3:** Major smart safety equipment with installation type.

Installation Equipment		Fundament and Method of Operation	Main Function
Mobile closed-circuit television (CCTV)	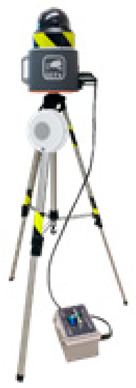	- LTE type - Real-time transmission of the video to the integrated control system - 72 h eternal battery, 2 megapixels, optical 20× zoom, 360°. - Detection of flames or smoke on CCTV associated with AI	- No limitation for the installation place - Real-time video check - Hazardous area approach detection - Detect wearing a helmet fire detection - Sending notifications to managers/workers
Hazardous gas detector	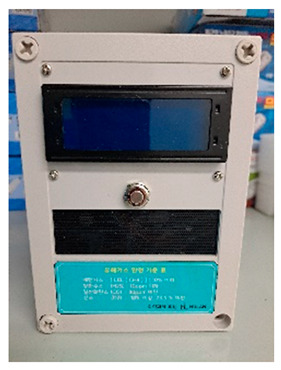	- BLE, long range (LoRa) type - Checking of temperature and humidity and various gas concentration detection. - Identification of CO, O2, H2S, CH4. - Determining the concentration of oxygen, carbon monoxide, methane, and hydrogen sulfide in a confined space - Self-warning alarm function	- Preliminary dentification of worker suffocation and harmful gas leakage - Noticing in the case of exceeding the standard value - Providing real-time metric recording
Structure Displacement/ tilt Sensor	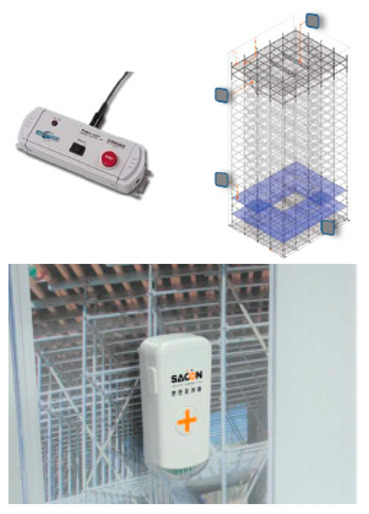	- LoRa, BLE type - Detection of displacement or inclination with three-axis acceleration - Alarming when tilting more than 0.5° occurs - Warning regarding tilt displacement and impact in conjunction with danger alarm - Utilize gyro/accelerometer sensor	- Measuring the displacement or tilt of temporary structure - Real-time measurement and recording of the above criteria and alarm caution - Zero-point installation for each structure
Opening open sensor	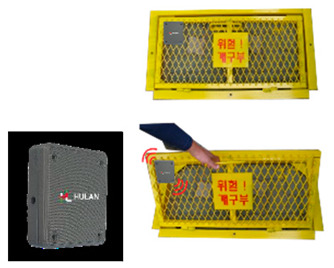	- LoRa type - Warning notification when opening a cover or approaching the surrounding area	- -Whether/number of openings are opened - Opening time & location
Heavy object drop alarm	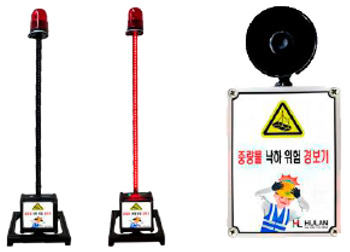	- Radio Frequency (RF) type - Installation at a place with a risk of falling - Controlling ON/OFF with remote control	- Alarming working with heavy objects
Thermal imaging camera	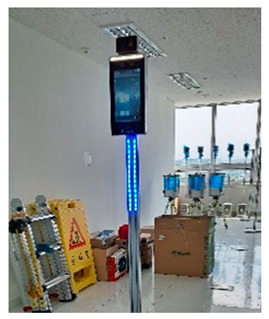	- Temperature check by face recognition. - FHD 1920 × 1080 resolution and sound alarm function in case of fever.	- Temperature check - Thermal image
Mobile integrated control system	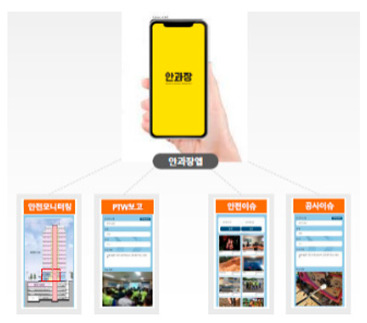	- BLE, LTE type - Information of smart safety equipment status & location, weather/air quality, danger alert	- Worker output management - Announcement - Operator emergency call function - Hazardous gas monitoring - Site equipment layout
PC integrated control system	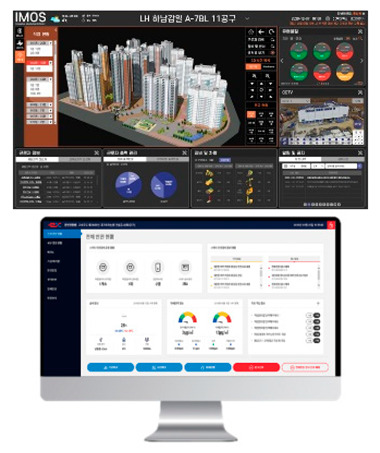	- Obtaining information by communication between the web site and central server. - If it is based on a smartphone, data transmitted and received with SCSTs are transmitted to the central server using its own LTE. - If there is no internet function of its own, the data transmitted and received between the SCSTs and the beacon is transmitted to the central server using Wifi or LoRa gate. - Necessary information for real-time status, deployment, etc., for SCSTs can be edited and used for the convenience of the administrator.	- Information of worker location, hazardous gas - Real-time video - Flow of heavy machinery - Worker output - Announcement

**Table 4:** Site information and installation equipment.

Type		A Site	B Site	C Site
Site information	Building type	Commercial building	Administrative agency	Sewage treatment plant
	Construction cost (million dollar)	4.2	10	5.4
	The number of workers	20	40	20
	Process rate	55%	28%	8%
	The number of floors	12th floor	2nd basement floor—5th floor	3rd Floor
Equipment type	Integrated control system	1	1	1
	Location sensor	23	11	11
	Fall risk area access (FRAA) sensor	9	2	2
	Heavy equipment access (HEA) sensor	3	3	1
	Mobile CCTV	1	1	2
	Hazardous gas detector	1	1	1
	Structure displacement sensor	2	2	2

**Table 5:** Location sensor data for one day for A site.

IN	OUT	Name	Type	Area	Location	Note
06:40:38		000	entry	Gate 2	QR reader	start work
07:05:14	07:12:02	000	general	F5	2	work path
07:28:21	07:28:56	000	general	F4	2	
07:28:56	07:29:38	000	general	F3	2	
07:29:38	07:30:15	000	general	F2	1	
...	...	...	...	...	...	-
11:30:27	11:31:25	000	entry	Gate 2	QR reader	lunch (15 min
11:46:05	11:46:27	000	general	F1	1	
11:48:06	11:49:10	000	general	F3	1	no break time
11:49:10	11:49:51	000	general	F5	2	
...	...	...	...	...	...	-
16:20:54	16:22:56	000	general	F2	1	work
16:22:56	16:23:58	000	general	F3	1	
16:23:58	16:23:58	000	general	F4	2	
16:29:43	16:31:45	000	general	F5	2	
16:31:45	16:31:45	000	general	Gate 2	QR reader	leave work

**Table 6:** Recommendations of SCSTs.

Equipment and Sensor	Necessity	Cost Effectiveness	Usability	Convenience	Scalability	Total Score
Location sensor	△	○	△	○	○	○
FRAA sensor	○	○	○	○	○	○
Mobile CCTV	○	X	○	○	○	○
Hazardous gas detector	○	○	△	○	X	△
HEA sensor	○	○	○	△	△	○
Structure displacement Sensor	○	○	○	○	X	○
Integrated control system	○	X	○	△	○	△

Note. ○: high, △: medium, X: low.

## Data Availability

The data presented in this study are available upon request from the corresponding author.
